# Pro- and Antioxidant Activity of Three Selected Flavan Type Flavonoids: Catechin, Eriodictyol and Taxifolin

**DOI:** 10.3390/ijms17121986

**Published:** 2016-11-26

**Authors:** Vladimir Chobot, Franz Hadacek, Gert Bachmann, Wolfram Weckwerth, Lenka Kubicova

**Affiliations:** 1 Division of Molecular Systems Biology, Department of Ecogenomics and Systems Biology, Faculty of Life Sciences, University of Vienna, Althanstrasse 14, A-1090 Vienna, Austria; gert.bachmann@univie.ac.at (G.B.); wolfram.weckwerth@univie.ac.at (W.W.); lenka.kubicova@univie.ac.at (L.K.); 2 Department of Plant Biochemistry, Albrecht-von-Haller Institut, Georg-August-Universität Göttingen, Justus-von-Liebig-Weg 11, D-37077 Göttingen, Germany; franz.hadacek@biologie.uni-goettingen.de

**Keywords:** flavonoids, redox chemistry, differential pulse voltammetry, deoxyribose degradation assay, Fenton reaction

## Abstract

The flavanol (±)-catechin shows an OH group but no 4-keto group on ring C (C3), and no conjugation between ring A and B. The related flavanone (+)-eriodictyol has a keto group on C4 but no 3-OH group on ring C. (+)-Taxifolin, another flavanone, has an OH on C3 and a keto group on C4 of the C ring. Deoxyribose degradation assay systems, with hydrogen peroxide and ascorbic acid either added or omitted, were performed in variants in which Fe(III) was added in a complex with ethylenediaminetetraacetic acid (EDTA). In combination with differential pulse voltammetry (DVP), the specific redox-chemical contributions of the ring A *m-*dihydroxyl groups could be explored more specifically in addition to those of the traditionally investigated *o-*dihydroxyl groups of ring B.

## 1. Introduction

Flavonoids are very common phenolic secondary plant metabolites with antioxidant activities that are closely related to their redox activities and transition metal complex formation properties [1,2]. Structure–activity relationships have been repeatedly explored [3,4,5]. So far, a catechol (*o-*dihydroxy) or pyrogallol (trihydroxy) arrangement on ring B is regarded as essential for antioxidant activity because the required energy for their dissociation is lower compared to that of other hydroxyl groups on ring A or C [6]. The flavonoids with the catechol or pyrogallol moieties may also show pro-oxidant effects in some reaction conditions [7,8]. A 2,3-double bond in ring C further increases the antioxidant effects of ring B hydroxyl groups [3] by extending electron dislocation [4]. The combinatory effects of the 3-hydroxyl group on ring C with the double bond affect the scavenging activity of flavonoids [3,9]. A higher number of hydroxyl groups in the flavonoid molecule increases not only the antioxidant [7,10] but also pro-oxidant activity [7] because their redox potentials are shifted into the cathodic direction [10]. A substitution of the hydroxyl groups of ring B or C, however, decreases the antioxidant activity [4,9]. The hydroxyl groups on ring B are usually attacked first by oxidative agents due to their low electrochemical redox potentials [10]. Conversely, the *m-*position of ring A hydroxyl groups causes their antioxidant properties to be lower than that of the *o*-dihydroxyl groups [11]. In addition, the mesomeric effect of the 4-keto group on ring C further contributes to this decrease [2]; higher electrochemical redox potentials are also required for their oxidation [12,13,14,15].

**Figure 1:**
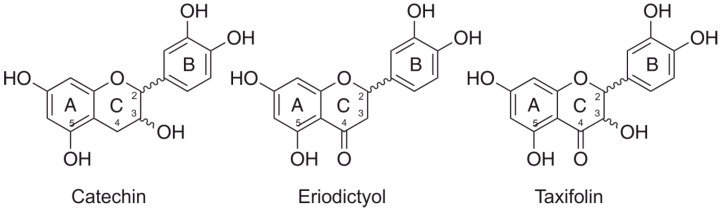
Structures of the flavan-type flavonoids that were included in the study.

The flavanol catechin (Figure 1) is one of the best-studied flavonoids. Its redox activity is especially interesting in the context of various biological effects [16,17,18,19]. Bais et al. formulated a hypothesis about the phytotoxicity of catechin that is exuded by the roots of *Centaurea stoebe*, an invasive spotted knapweed in North America [20]. An allelopathic and phytotoxic mechanism is attributed to the production of reactive oxygen species (ROS) in root tissues of competing plants [20]. By contrast, we found that catechin had no phytotoxic effects, but only weak pro-oxidant and strong antioxidant effects [16]. Catechin is also well-known for its cardio- [21] and neuroprotective [22] activities that depend on its antioxidant properties. Both the antioxidant [16,23,24,25] and pro-oxidant [16,23,26,27] effects vary due to the experimental conditions that were used in the respective studies.

The existing controversy in reports about the pro- and antioxidant activities of catechin motivated us to investigate its chemical, milieu-dependent redox properties. The flavanol (±)-catechin was compared with two structurally-related flavanones, (+)-eriodictyol and (+)-taxifolin, both with different oxygen functions on ring C, the first one with a keto group on C4 and the second one with an additional hydroxyl group on C3 (Figure 1). All three substances lack a 2,3-double bond on ring C; the ring A and B systems are not conjugated [28].

Traditionally, flavonoid redox properties have been evaluated by various voltammetric methods, such as cyclic [10,29], square-wave [30] and differential pulse [12,31] voltammetry. These technologies have been used in several studies as indicators for possible antioxidant activities, both for single compounds and for mixtures [32,33,34,35]. The voltammograms of phenols show one or more peaks. Their positions at specific potentials characterize the redox reactions on the electrode surface [36].

In an attempt to identify a practicable as well as informative combination of electrochemical and redox chemical methods, which would allow the study of the electrochemical and redox chemical contributions of two or more isolated aromatic ring systems within more complex molecules, we used a combination of differential pulse voltammetry and variants of the deoxyribose degradation assay [16,37]. This assay uses 2-deoxy-d-ribose as a detection molecule and is utilized to assess the antioxidant properties of various compounds or compound mixtures [38]. 2-Deoxy-d-ribose is degraded to thiobarbituric acid reactive species (TBARS) by the hydroxyl radical (^•^OH) formed in the Fenton reaction [37].

Fe(II) + H_2_O_2_→Fe(III) + HO^–^ + ^•^OH

Iron is added in complex with ethylenediaminetetraacetic acid (EDTA), allowing the study of the reduction activities of tested compounds. Ascorbic acid reduces Fe(III) to Fe(II) in the classical reaction mixture. In its absence, the capability of the flavonoid to reduce Fe(III) can be evaluated [39]. Hydrogen peroxide (H_2_O_2_) is an important reactant of the Fenton reaction. Likewise, its absence facilitates research into whether the flavonoid—given that the duration of the assay is extended from 1 to 16 h—can reduce molecular oxygen to superoxide anion radicals that, by subsequent dismutation, generate sufficient H_2_O_2_ levels. The detailed advantages of the different variants of the assay are summarized by Chobot [37].

## 2. Results

### 2.1. Differential Pulse Voltammetry

**Figure 2:**
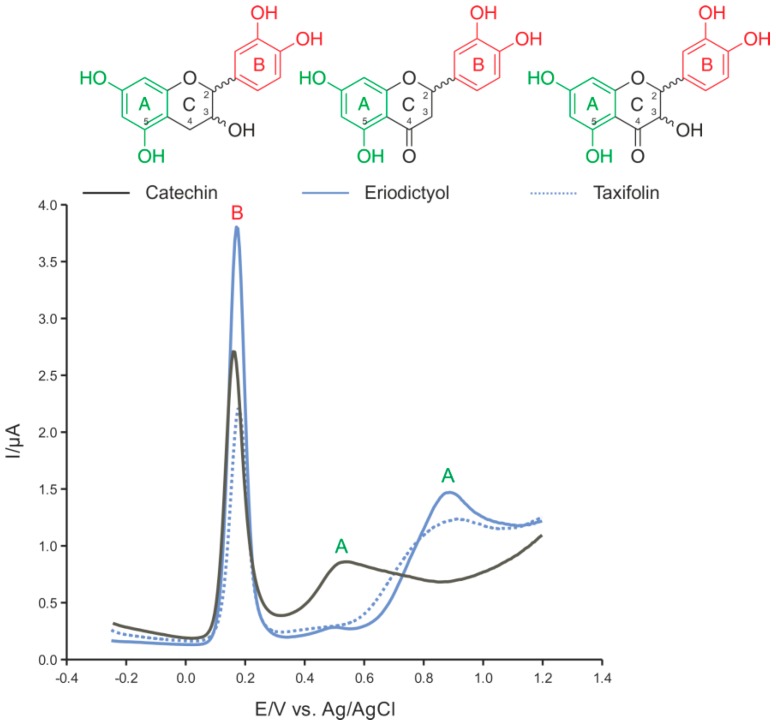
Differential pulse voltammograms of the tested flavans: catechin, eriodictyol, and taxifolin.

The voltammograms of the tested flavans showed two prominent peaks (Figure 2). The first represented the anodic oxidation of *o*-dihydroxyl groups of ring B; the second, the oxidation of the hydroxyl groups of ring A [13,40].

The general notion is that the antioxidant effects of flavonoids are due to their redox and chelation activities [2,4,41]. Several studies have shown that the number and arrangement of the phenolic hydroxyl groups affects the efficacy of their antioxidant activity [4,41]. Compared to taxifolin and eriodictyol, catechin shows a peak for ring A at a lower potential. By contrast, the ring B hydroxyl groups’ *E*_p_ values are very similar for all three tested flavans (Table 1, Figure 2).

The *o-*dihydroxyl groups of ring B are usually the initial target of oxidants [2,4]. Their electrochemical redox potential (*E*_p_) is lower than that of the *m*-dihydroxyl groups of ring A [13,40]. The negative mesomeric effect of the 4-keto group on ring C affects the *E*_p_ of the ring A hydroxyl groups [2] (see eriodictyol and taxifolin). The peak currents corresponding to the oxidation of *m*-dihydroxyl groups of ring A are lower and the peak is broader compared those of the *o-*dihydroxyl groups of ring B. In general, the peak current depends on many factors, such as the rate of diffusion and the number of exchanged electrons, among others. The shapes of the ring A peaks and their broadness suggest a possible anodic electrochemical oxidation process followed by a chemical reaction (EC process) [36].

### 2.2. Deoxyribose Degradation Assay

**Figure 3:**
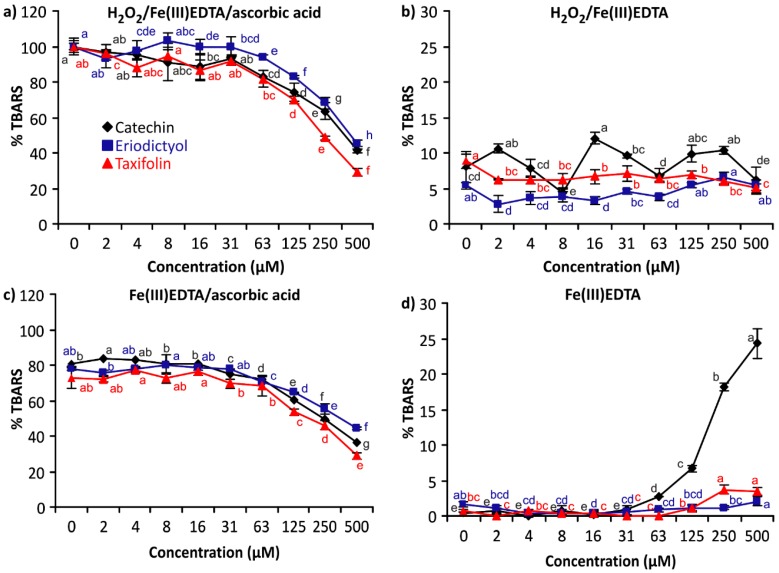
Thiobarbituric acid reactive species (TBARS) production in the reaction mixtures of the deoxyribose degradation assay: (**a**) H_2_O_2_/Fe(III)EDTA/ascorbic acid (1 h incubation); (**b**) H_2_O_2_/Fe(III)EDTA (1 h incubation); (**c**) Fe(III)EDTA/ascorbic acid (16 h incubation); and (**d**) Fe(III)EDTA (16 h incubation) (100% = TBARS of the control reaction mixture of the assay variant H_2_O_2_/Fe(III)EDTA/ascorbic acid). Error bars show standard deviation (SD) of three replicates; letters (a–h) indicate levels of significance (95% Duncan); EDTA = ethylenediaminetetraacetic acid.

Electrochemical redox properties also determine the activities of catechin, eriodictyol and taxifolin in the deoxyribose degradation assay. Depending on the respective assay system, the presence of the flavonoids in the reaction mixture either slows or speeds up the generation of TBARS (formed after the destruction of deoxyribose by ^•^OH). Figure 3 shows the TBARS development obtained for the three flavans for all tested concentrations in all assay variants. All tested flavans showed antioxidant activity in all assay variants where ascorbic acid was added to the reaction mixture. Taxifolin was the strongest antioxidant, followed by catechin and eriodictyol. Generally, the antioxidant activity increased with the concentration of the flavonoids.

In the system H_2_O_2_/Fe(III)EDTA, all three flavonoids did not affect the TBARS concentrations compared to the control, despite the fact that some values significantly differed from the control (0 conc. of flavonoids), but this difference was considered to be irrelevant (<5% TBARS). Only when both H_2_O_2_ and ascorbic acid were omitted, catechin showed a weak pro-oxidant effect in the highest three concentrations tested. Again, significant differences from the control were not accompanied by relevant changes in the TBARS concentration. Eriodictyol and taxifolin demonstrated no pro-oxidant activity within the tested concentration range.

## 3. Discussion

Natural phenolic compounds such as flavonoids scavenge ROS by proton-coupled electron transfer [42]. By this transfer, the phenolic compounds are oxidized to semiquinones or quinones. Firuzi et al. [10] noted a good correlation between the electrochemical redox potential of the hydroxyl groups of ring B with antioxidant activities in the ferric ion reducing antioxidant power (FRAP) assay, which explores the capability of the tested substance to reduce ROS or free radicals to terminate chain radical reactions. Similarly, Yang et al. [3] found a correlation between the half-wave electrochemical redox potential of the first flavonoid redox peak with activity in a microsomal lipid peroxidation assay. Both author groups did not explore the redox potential effects of the hydroxyl groups of ring A on the antioxidant effects of the flavonoids. However, Tsimogiannis et al. suggested the role of ring A in ROS scavenging and cotton oil stabilization [11]. To explore this in detail, we investigated the effects of the ring A system on the redox properties of catechin in comparison to two other structurally similar flavans in several variants of the deoxyribose degradation assay.

Formally, the deoxyribose degradation assay systems are characterized by the presence or absence of ascorbic acid in the reaction mixture. Ascorbic acid reduces Fe(III) to Fe(II) that again can reduce H_2_O_2_ to ^•^OH (Fenton reaction) or, in the absence of other possible reactants, molecular oxygen to superoxide anion radicals (O_2_^•−^), which further dismutate to H_2_O_2_. Contrary to a radical-scavenging antioxidant activity, this is a pro-oxidative effect [37]. Similarly to ascorbic acid, flavonoids can also act as reducing agents in the reaction mixture. Accordingly, ^•^OH that arise from the Fenton reaction may be scavenged by the flavonoids, depending on their concentration. Preferably, ^•^OH reacts with the *o-*dihydroxyl groups of ring B.

If ascorbic acid is absent in the reaction mixture, only traces of TBARS are produced in the reaction mixture. This does not change when either eriodictyol or taxifolin is added. Only catechin affected the TBARS concentration in the Fe(III)EDTA complex reaction mixture. Catechin increased the TBARS concentration when compared to the TBARS levels when catechin is not added to the reaction mixture (Figure 3d). This pronounced pro-oxidative effect, however, requires longer than 1 h to develop because it depends on the diffusion of oxygen into the reaction mixture. Flavonoids with *o*-dihydroxyl groups on ring B are known to generate H_2_O_2_ by reducing molecular oxygen to O_2_^•−^ [43,44]. However, the catechol moiety is not the only prerequisite because eriodictyol and taxifolin proved to be non-pro-oxidative in the same assay.

The electrochemical properties of catechin, eriodictyol and taxifolin provided additional insights to interpret their contrasting redox activities and, in particular, their electrochemical redox potentials, especially that of the ring A hydroxyl groups. According to our expectations based on the deoxyribose degradation assay results, the differential pulse voltammogram of catechin revealed that the *E*_p_ of the ring A hydroxyl groups was lower than that of eriodictyol and taxifolin (less efficient reducing agents). This indicated that the ring A hydroxyl groups of catechin were more reductive than those of eriodictyol and taxifolin. The pro-oxidative effect of catechin suggests that ring A and ring B may both act as independent reduction units. Only in the case of catechin, however, do the two ring systems develop a reduction force strong enough to start the Fenton reaction in the initial absence of H_2_O_2_. In addition, hydroxylation reactions of phenolic compounds during the anodic electrochemical processes have been observed [45]. Although a direct relation between reactions on the electrode surface and those in the reaction solution of the deoxyribose degradation assay and cell protoplast is difficult to determine, the formation of further substances with redox activity during the oxidation of flavans with ROS is suggested. Therefore, in the reaction mixture, a complex equilibrium between ROS formation and their scavenging may have developed. Furthermore, this equilibrium is additionally affected by the reaction products of the flavans with ^•^OH which oxidize the phenols to semiquinones and quinones, but also form hydroxyl derivatives as well as flavan oligomers [28,46,47]. These flavan reaction products between flavans and ^•^OH may also show pro- or antioxidant properties.

The obtained results point to the fact that the antioxidant or pro-oxidant activity of the flavans depends not only on the number of phenolic hydroxyl groups, but also on other factors. The presence of the 4-keto group could especially play a key role. This keto group on C4 decreases the possible pro-oxidant effects of the flavans. In addition, electrochemical and deoxyribose degradation data provide support for the postulated redox independency of ring A hydroxyl groups in flavonoids that lack a 2,3-double bond in ring C (flavan-type). To our knowledge, this aspect was not pointed out specifically in previous studies, despite the fact that the *E*_p_ values of ring A differ between the three tested flavans (mesomeric effect of the keto group on C4 of ring C). This illustrates the complex chemical behavior of specific combinations of functional groups in larger molecules. They reflect complex systems with feed forward and feedback effects. Functional groups affect chemical reactions, not only by their presence and absence but also by the specific interactions between them that change with their chemical environment. This explains why unambiguous predictions of the effects of flavonoids and similar secondary metabolites in living tissues are nearly impossible [16,48]. It would be advantageous to apply this methodological approach to studies of similar interest.

## 4. Materials and Methods

### 4.1. Chemicals

Hydrogen peroxide, 2-deoxy-d-ribose, and (+)-taxifolin (referred to as taxifolin throughout the text) were obtained from Fluka (Buchs, Switzerland). (±)-Catechin (referred to as catechin throughout the text), (+)-eriodictyol (referred to as eriodictyol throughout the text) and all other chemicals and organic solvents were purchased from Sigma-Aldrich (St. Louis, MO, USA). All chemicals and organic solvents were of analytical grade. The water was of Milli-Q quality (Milli-Q Advantage A10 System, Milllipore SAS, Molsheim, France).

### 4.2. Differential Pulse Voltammetry

Voltammetric curves were recorded in a three-electrode system, µAutolab PGSTAT type III (EcoChemie Inc., Utrecht, The Netherlands). The working electrode was a glassy carbon electrode of 3 mm diameter, an Ag/AgCl (saturated KCl) electrode was used as reference, and platinum wire as a counter electrode. The glassy carbon electrode was cleaned with methanol and water and polished before every measurement. The effective scan rate of the voltammetry was 21 mV·s^−1^, modulation time was 0.05 s, and modulation amplitude was 25 mV, and the scan potential was from −0.250 to +1.200 V. 1 mL of 1 mM solution of the tested flavan in degassed methanol was mixed with 9 mL of the degassed buffer (0.1 M phosphate buffer pH 7.4). All electrolyte solutions were degassed by argon for 10 min. The measurements were performed under argon atmosphere at room temperature (20 °C).

### 4.3. Deoxyribose Degradation Assay

The various systems of the assays follow procedures, the detailed reaction mechanisms of which are described elsewhere [37]. The flavonoids were dissolved in different concentrations (0−500 µM) in an aqueous KH_2_PO_4_/KOH buffer solution (30 mM, pH 7.4); to 125 µL of this solution, 25 µL of a 10.4 mM 2-deoxy-d-ribose solution in the same buffer system and 50 µL of Fe(III)EDTA solution (50 µM) were added. Those 50 μL contained 52 μM EDTA dissolved in buffer, which was premixed with the aqueous FeCl_3_ solution (1:1 *v*/*v*). Furthermore, 25 µL of 10.0 mM H_2_O_2_ in water and 25 µL of 1.0 mM ascorbic acid in the buffer were added in the case of H_2_O_2_/Fe(III)EDTA/ascorbic acid assay variant. In the other deoxyribose degradation assay systems, H_2_O_2_ or ascorbic acid was replaced by water or buffer respectively. The temperature during incubation was 27 °C. The assay systems with H_2_O_2_ were incubated for 1 h; the systems without H_2_O_2_ were incubated for 16 h. After reaction with thiobarbituric acid and subsequent extraction of the pink pigment with 1-butanol, oxidative degradation products of 2-deoxy-d-ribose (TBARS) were determined photometrically at 532 nm. The positive control (100% TBARS) was the H_2_O_2_/Fe(III)EDTA/ascorbic acid reaction mixture without the test compound. The blank contained the full reaction mixture except 2-deoxy-d-ribose and also was determined in each performed experiment series. Assays were performed in triplicate.

### 4.4. Statistics

One-way ANOVA with 95% Duncan’s multiple range test was used to determine significant differences between concentrations in the deoxyribose degradation assay.

## Figures and Tables

**Table 1:** Electrochemical variables for flavan ring A and ring B hydroxyl groups extracted from differential pulse voltammogram data (mean ± SD of three measurements)

Flavan	Catechin	Eriodictyol	Taxifolin
**Ring A**			
*E*_p_ (V)	0.518 ± 0.005	0.873 ± 0.003	0.851 ± 0.016
*I*_p_ (nA)	339.8 ± 34.4	707.0 ± 5.2	399.7 ± 35.0
**Ring B**			
*E*_p_ (V)	0.160 ± 0.002	0.170 ± 0.000	0.179 ± 0.003
*I*_p_ (nA)	2144.0 ± 564.1	3447.0 ± 272.9	2141.0 ± 151.2

*E*_p_, electrochemical redox potential (vs. Ag/AgCl) at peak maximum; *I*_p_, peak current at peak maximum.
